# Chemical Conservation of Paper-Based Cultural Heritage

**DOI:** 10.3390/molecules30010122

**Published:** 2024-12-31

**Authors:** Yueer Yan, Yi Tang, Yuliang Yang

**Affiliations:** 1 Institute for Preservation and Conservation of Chinese Ancient Books, Fudan University Library, Fudan University, Shanghai 200433, China; 2 Department of Chemistry, Fudan University, Shanghai 200433, China; 3 Department of Macromolecular Science, Fudan University, Shanghai 200433, China

Paper-based cultural heritages, represented by ancient books, archives, calligraphy, and paintings, recorded the development of human civilization. However, they are inevitably deteriorated during their long-term preservation, hence requiring intensive care to protect and inherit them. Chemists can play crucial roles in this mission, considering the interdisciplinary nature of the field of heritage science. The relationship between heritage science and applied chemistry is particularly close when facing the problems of paper aging, where intrinsic and extrinsic factors are entangled to form convoluted degradation mechanisms. Since the public awareness of cultural heritage protection is increasing, an ever-growing effort has been devoted to the preservation and conservation of paper artifacts.

This Special Issue successfully collected the important contributions in the field of heritage conservation. Contributions include studies on the properties and degradation mechanisms of carriers of cultural heritages, the characteristics of adhesives commonly used in mounting and restoration, and the various materials employed in the cleaning, deacidification, and reinforcement of paper-based artifacts. By carrying out comprehensive research on paper-based cultural heritage, this Special Issue aims to provide a deeper understanding of the mechanisms involved in the degradation and preservation of paper artifacts and offers practice insights into more efficient and sustainable conservation methods.

Paper has been focused on its unique physical and chemical properties when used as the carrier of paper-based cultural heritage. Japanese *kozo* paper is commonly used as the first back lining paper of hanging scrolls, which plays a crucial role in supporting the main paper with a painting or a calligraphy on it. This lining paper is often dyed and adhered during the mounting process and, therefore, a high wet tensile strength is essential. Han et al. investigated the effects of hemicellulose (especially glucuronoxylan) on the wet tensile strength of *kozo* paper produced in different cooking conditions (contribution 1). The wet tensile strength of paper was evaluated using the Finch device, and the content of glucuronoxylan in fiber was analyzed by a gas chromatographic method. By analyzing the glucuronoxylan content and wet tensile strength of various *kozo* paper samples, the authors revealed that papers with higher glucuronoxylan content exhibited stronger wet tensile strength. Then, they studied the effects of cooking time and the type and concentration of alkali on the glucuronoxylan content of paper under varying cooking conditions. The results indicated that the cooking time had minimal impact, while using alkali with weaker alkalinity and lower concentration resulted in higher glucuronoxylan retention within the fibers and stronger wet strength of *kozo* paper. Furthermore, they added the extracted glucuronoxylan into the *kozo* pulp under identical cooking conditions. The as-prepared papers showed a proportional increase in wet tensile strength with increasing glucuronoxylan content, indicating that glucuronoxylan itself contributes significantly to the wet tensile strength of *kozo* paper. This work provides a guideline for the production of *kozo* paper with an expected wet tensile strength and contributes to the preservation and conservation of cultural heritage with good paper quality.

The existence of hemicellulose not only affects paper’s wet tensile strength, but also influences the long-term stability of paper. Zhu et al. studied the structure and properties of Chinese bamboo paper during the aging process and focused on the influence of hemicellulose and lignin on paper durability (contribution 2). It was found that the mechanical properties of bamboo paper with high lignin and hemicellulose content exhibited three stages during dry-heat aging: initial plateau, rapid decline, and second plateau as the degree of polymerization (DP) of cellulose decreased. The contents of cellulose and hemicellulose decreased while lignin remained relatively stable. By analyzing the changes in microstructures during the aging process, such as hydrogen bonds, hornification, pore structure, and crystallinity, the aging mechanisms of bamboo paper were proposed. The decrease in DP of cellulose led to fiber embrittlement, but the enhancement of hydrogen bonds and the hornification process could partially compensate for this effect, thereby maintaining the mechanical properties of paper during the initial plateau stage. When the DP of cellulose fell below the critical value, the deep cleavage of cellulose chains and the release of a large number of degradation products caused a decrease in cellulose content and an increase in paper acidity, resulting in a sharp decline in paper mechanical properties. The existence of hemicellulose and lignin might contribute to the stability of paper structure by inhibiting cellulose aggregation, thus, forming a second plateau stage. This research not only improves the comprehension of the relationship between the structural properties and degradation mechanisms of handmade papers but also provides a scientific foundation for the production of handmade paper with better durability.

Similarly to paper, palm leaves are used as writing and recording materials, derived from plant resources. Palm leaves served as a popular literary medium in South and Southeast Asia before the widespread use of paper, which played an invaluable role in the development of human civilization. Studies on the mechanical properties of palm leaf manuscripts under different environmental conditions are of great significance to understand the material characteristics, aging mechanisms, and preventive conservation methods of these manuscripts. Zhang et al. investigated the short-term and long-term effects of relative humidity (RH) on the mechanical properties of palm leaf manuscripts (contribution 3). In their experiments, dynamic vapor sorption (DVS) was used to measure the hygroscopic behavior of palm leaves in response to different humidity conditions, and a thermomechanical analyzer–modular humidity generator (TMA-MHG) was used to analyze the mechanical behavior (bending strength and bending modulus) of the samples at various humidity levels. The short-term study showed that the bending strength of the palm leaf samples decreased significantly with the increase in humidity, while the bending modulus firstly increased and then decreased. A stable RH could slow down the variations in the mechanical properties, thereby preventing the degradation of palm leaf materials. The long-term results indicated that the prolonged exposure to either extremely dry or humid environments could seriously affect the mechanical properties of palm leaves, which was detrimental to their preservation. Samples aged at 50% RH had no visible damage, and the mechanical properties and chemical structures were well preserved, suggesting that 50% RH should be an optimal humidity condition for preserving palm leaf manuscripts. This study provides a comprehensive understanding of the mechanical behaviors of palm leaf manuscripts and supports the development of long-term preservation and preventive conservation of these priceless manuscripts.

For paper-based cultural heritage, the use of adhesives plays a crucial role, especially in artwork mounting and paper sizing. Wheat starch has been a common adhesive material used in the mounting of calligraphies and paintings for over a millennium. Since the naturally derived starch gel has certain limitations, such as relatively low viscosity and easy retrogradation, Chinese restorers discovered that the combination of wheat starch with potassium alum (PA) offered promising improvements in the adhesive strength and stability of starch gel. Zhao et al. investigated the effects of varying PA contents on the thermal, rheological, structural, and adhesive properties of wheat starch gels (contribution 4). The results indicated that the incorporation of PA elevated the gelatinization temperature and enthalpy of the starch gels, with a maximum leached amylose and swelling power observed at a specific wheat starch to PA weight ratio of 100:6. Rheological measurements and scanning electron microscopy (SEM) observations corroborated this phenomenon. Fourier transform infrared spectroscopy (FTIR) analysis showed an enhanced short-range molecular order in the gels with PA. Mechanical experiments revealed that the binding strength of the starch gels increased with higher PA concentrations and decreased significantly after the aging process. Therefore, it is advisable to strictly control the concentration of PA in starch gels to balance the enhanced rheological and adhesive properties with the minimization of negative impacts on the degradation and color change of paper relics. This study provides a comprehensive understanding of the effects of PA on starch gels and offers practical insights for the restoration of valuable cultural artifacts.

Starch gel and alum gelatin are also widely used as natural sizing agents for paper. However, natural starch has a poor affinity with fibers and a tendency to detach, and gelatin alum poses a threat to the durability of paper. Hence, studies on efficient and harmless agents for paper sizing are still required in the conservation of cultural heritage. Wu et al. investigated the use of soymilk as a sizing agent for Xuan paper, focusing on its impact on paper properties and long-term stability (contribution 5). They prepared different concentrations of soymilk and characterized its pH, viscosity, particle size, and zeta potential. The soymilk was applied to Xuan paper, and the soymilk-sized paper had improved hydrophobicity, enhanced mechanical properties, and unique chromaticity compared to unsized paper. These characteristics are attributed to the papillae on the surface of Xuan paper, the folding of the soy protein, and the hydrogen bond interactions between the soy protein and paper fibers. The soymilk-sized paper also showed a higher initial pH, which declined more slowly than alum gelatin-sized paper, suggesting better acid resistance. After accelerated aging, the mechanical strength and color fidelity of paper deteriorated, and the expected lifespans of paper decreased with the increasing soymilk concentration. Compared with paper sized with alum gelatin, the soymilk-sized paper showed better hydrophobicity and durability. This study highlights the potential benefits and challenges of using soymilk as a paper sizing agent and aids the selection of proper materials for the mounting and conservation of paper artifacts.

Additionally, research on adhesives has been expanded to the protection of wooden heritage objects. Wood composed of cellulose, hemicellulose, and lignin is a natural material whose physical and chemical properties change during the preservation process, leading to varying degrees of degradation. Traditional adhesives used in conservation of wood artifacts have their limitations, particularly in terms of low adhesion, durability, and antimicrobial properties. Teri et al. investigated the synthesis and application of a novel adhesive for preserving wooden heritage objects. The adhesive is prepared by modifying fluorinated ethylene vinyl ether (FEVE) resin with Ag_2_O-decorated hydroxyl multiwall carbon nanotubes (Ag_2_O/OH-MWCNTS, MA) composites, and the prepared adhesive was named MAF (contribution 6). The resulting MAF adhesive showed enhanced thermal stability, higher viscosity, and effective bacteriostatic property compared to pure FEVE resin. The viscosity and adhesion strength of MAF increased with the increase in MA concentration, indicating that Ag_2_O nanoparticles acted as physical crosslinking agents within the FEVE resin. Compared with pure FEVE resin, the MAF adhesive had a lower mass loss rate after treatment at 800 °C and exhibited better thermal stability. The antibacterial activity of MAF is directly related to the concentration of Ag_2_O/OH-MWCNTS, with the significant inhibitory effect on *Trichoderma*, *Aspergillus niger*, and *white rot fungi*. The MAF adhesive shows promising prospects for use in wooden heritage artifacts, offering a novel approach for the rapid, environmentally friendly, and efficient preparation of composite adhesives with superior adhesive properties.

The restoration of paper artifacts is of great importance to ensure their longevity. However, traditional restoration methods are time-consuming and always contain multistep processes, which highlights the need for alternative methods that can provide a more effective, gentle, and sustainable approach to protect cultural heritages. One potential solution that has emerged in recent years is the use of ionic liquids. Ionic liquids are composed entirely of ions, which gives them unique properties and makes them suitable for paper cleaning and antimicrobial purposes. Croitoru et al. investigated the application of ionic liquids for restoring aged paper artifacts, focusing on their ability to clean, deacidify, and stabilize against ultraviolet (UV) radiation (contribution 7). The treated paper exhibited significant color enhancements, indicating a good cleaning ability of ionic liquids. Moreover, the selected ionic liquids displayed pronounced deacidification capabilities, leading to a neutral pH of paper. They also acted as potential UV stabilizers to minimize the yellowing and discoloration of paper caused by UV radiation. All these results demonstrate the potential use of ionic liquids as a novel and effective approach for the restoration of paper artifacts.

The deterioration of paper always causes its embrittlement and the loss of mechanical strength. Organosilane materials have emerged as promising candidates for paper reinforcement due to their dual advantages of organic and inorganic properties. Wu et al. introduced dodecyltrimethoxysilane (DTMS) into tetraethoxysilane (TEOS) to prepare the hybrid materials as reinforcement agents for paper relics (contribution 8). By incorporating DTMS with flexible long chains, they aimed to weaken the capillary forces and minimize the shrinkage during the sol–gel process of TEOS. The gel formed from the DTMS/TEOS hybrid material was transparent and crack-free, featuring a dense microstructure with excellent thermal stability. When applied to bamboo paper, the hybrid material combined with the paper fibers through the sol–gel process and polymerized into a network structure that enveloped the paper surface or penetrated between the fibers. The surface of the treated paper displayed excellent hydrophobic properties, with no significant changes in appearance, color, or air permeability. The tensile strengths of the treated bamboo paper improved significantly, indicating the efficient reinforcement of paper by DTMS/TEOS. The developed hybrid material demonstrates a positive reinforcement and preservation effect on paper-based cultural relics. The advantages of ease of preparation, low cost, and wide accessibility make DTMS/TEOS a promising choice for future applications in paper conservation.

In addition to brittleness, the aging and degradation of paper also lead to other problems such as yellowing, discoloration, and acidification. Hence, new restoration materials are emerging for the multifunctional protection of paper-based cultural relics. Wang et al. synthesized low-dimensional and mildly alkaline magnesium carbonate hydrates (MCHs) for the deacidification, strengthening, and flame retardance of paper relics (contribution 9). The one-dimensional (1D) whisker and two-dimensional (2D) nanosheet MCHs are prepared through a simple supersaturation control method without the need for organic additives. The results indicated that MCHs with mild alkalinity exhibited good safety, showing minimal color change to the paper and little effect on alkali-sensitive pigments. Both 1D and 2D MCHs effectively neutralized the acidity in the paper and maintained a long-term alkaline reserve. Due to the structural properties of two kinds of MCHs, 1D MCHs showed better performance than 2D MCHs in enhancing the mechanical strength of paper and providing a flame-retardant effect. The ability to control the microstructure of MCHs allows for the design of materials with specific properties for different applications. This study also gives a valuable insight into the development of new conservation materials and expands the understanding of functional deacidification and protection mechanisms.

The use of composite materials provides more functions and better performance for the conservation of aged paper. Nanocellulose with natural paper compatibility has become a promising nanomaterial for paper repairing. The combination of nanocellulose with alkaline nanoparticles has been widely applied for the simultaneous reinforcement and deacidification of paper. Mou et al. prepared magnesium oxide (MgO) nanoparticles carried by aminosilaned bacterial cellulose (KH550-BC) for the restoration of aged paper (contribution 10). The existence of MgO in KH550-BC enhanced the paper pH and provided an alkaline reserve to counteract future acidification, while KH550-BC improved the mechanical properties of paper, including tear index, tensile index, and folding endurance. The hydrogen bond interactions between KH550-BC and paper fibers as well as the formed network structure of BC enhanced the paper strength. The hydrophobicity of paper is maintained after treatment, preventing damage from moisture and humidity. Artificial aging tests demonstrated that KH550-BC/MgO-treated paper exhibited superior resistance to acidification, mechanical degradation, and yellowing compared to untreated paper. The results of this work indicate that KH550-BC/MgO is an effective deacidification and reinforcement material for paper-based cultural heritage.

In conclusion, research about the chemical conservation of paper-based artifacts, especially focusing on the properties and applications of carriers, adhesives, and restoration materials, holds significant importance in the field of heritage science. Through continuous technological innovation and interdisciplinary integration, we are poised to discover more effective conservation solutions for the inheritance and protection of precious cultural heritage.

